# Supplementary material for: Electrophysiological markers for anticipatory processing of nocebo-augmented pain
Source: PLoS One. 2023 Jul 26;18(7):e0288968. doi: 10.1371/journal.pone.0288968 (PMC10370880; doi:10.1371/journal.pone.0288968)
Supplement: Attachment — Submitted filename: Blythe et al response letter.docx [file pone.0288968.s001.docx]

June 09, 2023

Dear Prof. De Pascalis,

Thank you for the opportunity to revise and resubmit our manuscript "Electrophysiological markers for anticipatory processing of nocebo-augmented pain”*.* We have revised the manuscript based on the reviewer’s suggestions. Below, please find an itemized series of responses to reviewer comments. All revisions made to submitted documents can be seen in tracked changes. The noted sections and paragraph numbers provided below indicate the location of specific revisions when the manuscript is viewed with all markup and track changes on.

Sincerely on behalf of all authors,

Joseph Blythe

Response to Reviewer Comments:

**Reviewer 1**

*Overall, the authors do a thorough job describing their study which address a gap in knowledge.
There are some sections that may be trimmed for brevity and some grammatical errors but overall the paper brings interesting findings of how top down processing plays into sensory perception.*

**Response**

We thank the reviewer for their compliments on the manuscript, and for highlighting the need to improve the brevity of the writing and to correct grammatical errors. We have thoroughly revised the manuscript with an emphasis on these points, improving brevity and correcting errors in grammar throughout all sections of the manuscript.

Response to Journal Comments:

1. *Please ensure that your manuscript meets PLOS ONE's style requirements, including those for file naming. The PLOS ONE style templates can be found at*

*https://journals.plos.org/plosone/s/file?id=wjVg/PLOSOne_formatting_sample_main_body.pdf and*

*https://journals.plos.org/plosone/s/file?id=ba62/PLOSOne_formatting_sample_title_authors_affiliations.pdf*

**Response**

We have updated the manuscript according to the above mentioned style guides.

2. *Thank you for stating the following in the Acknowledgments Section of your manuscript:*

*This research was supported by an NWO Vici grant (45316004) and an NWO Stevin award granted to A. Evers.*

*However, funding information should not appear in the Acknowledgments section or other areas of your manuscript. We will only publish funding information present in the Funding Statement section of the online submission form. 
Please remove any funding-related text from the manuscript and let us know how you would like to update your Funding Statement. Currently, your Funding Statement reads as follows:*

*This work was supported by a Dutch Research Council (NWO) Vici grant (#45316004) awarded to A.W.M. Evers. Website: nwo.nl. The funders had no role in study design, data collection and analysis, decision to publish, or preparation of the manuscript.*

*Please include your amended statements within your cover letter; we will change the online submission form on your behalf.*

**Response**

Thank you for catching this error. We would like for the updated funding statement to read as follows,

“*This work was supported by a Dutch Research Council (NWO) Vici grant (#45316004) and an NWO Steven award granted to A.W.M. Evers. Website: nwo.nl. The funders had no role in study design, data collection and analysis, decision to publish, or preparation of the manuscript.*”

3. *We note that you have stated that you will provide repository information for your data at acceptance. Should your manuscript be accepted for publication, we will hold it until you provide the relevant accession numbers or DOIs necessary to access your data. If you wish to make changes to your Data Availability statement, please describe these changes in your cover letter and we will update your Data Availability statement to reflect the information you provide.*

**Response**

Should the manuscript be accepted, we will gladly upload the data to the DataverseNL data archive and provide the necessary details to access the data.

4. *Please review your reference list to ensure that it is complete and correct. If you have cited papers that have been retracted, please include the rationale for doing so in the manuscript text, or remove these references and replace them with relevant current references. Any changes to the reference list should be mentioned in the rebuttal letter that accompanies your revised manuscript. If you need to cite a retracted article, indicate the article’s retracted status in the References list and also include a citation and full reference for the retraction notice.*

**Response**

We have checked the reference list for completeness and correctness. No retracted papers are cited.

**Additional editor comments**

I agree with the reviewer that the manuscript is well done and brings interesting findings of how top-down processing plays into sensory perception. I agree also agree that the manuscript should be shortened and The English revised by a native English speaker.

**Response**

*We thank the editor for the kind words on the manuscript and the advice for its improvement. We have shortened the manuscript wherever it was possible without removing relevant content, and had a native English speaker check for grammar and spelling errors.*

End of responses.
